# Omega-6:3 Ratio More Than Absolute Lipid Level in Diet Affects Associative Learning in Honey Bees

**DOI:** 10.3389/fpsyg.2018.01001

**Published:** 2018-06-19

**Authors:** Yael Arien, Arnon Dag, Sharoni Shafir

**Affiliations:** ^1^B. Triwaks Bee Research Center, Department of Entomology, The Robert H. Smith Faculty of Agriculture, Food and Environment, The Hebrew University of Jerusalem, Rehovot, Israel; ^2^Gilat Research Center, Institute of Plant Sciences, Agricultural Research Organization, Negev, Israel

**Keywords:** *Apis mellifera*, omega-6, omega-3, cognition, conditioning, nutrition

## Abstract

Floral pollen is a major source of honey bee nutrition that provides them with micro- and macro-nutrients, including proteins, fatty acids, vitamins, and minerals. Different pollens vary in composition, including in the essential fatty acids, alpha-linolenic acid (omega-3) and linoleic acid (omega-6). Monocultures, prevalent in modern agriculture, may expose honey bee colonies to unbalanced omega-6:3 diets. The importance of omega-3 in the diet for adequate learning and cognitive function, with a focus on suitable omega-6:3 ratio, is well documented in mammals. We have recently shown, for the first time in invertebrates, the importance of omega-3 in diets for associative learning ability in honey bees. In the current work, we examine the effect of the absolute amount of omega-3 in diet compared to the omega-6:3 ratio on honey bee associative learning. We fed newly emerged bees for 1 week on different artificial diets, which had lipid concentration of 1, 2, 4, or 8%, with omega-6:3 ratios of 0.3, 1, or 5, respectively. We then tested the bees in a proboscis-extension response olfactory conditioning assay. We found that both omega-6:3 ratio and total lipid concentration affected learning. The most detrimental diet for learning was that with a high omega-6:3 ratio of 5, regardless of the absolute amount of omega-3 in the diet. Bees fed an omega-6:3 ratio of 1, with 4% total lipid concentration achieved the best performance. Our results with honey bees are consistent with those found in mammals. Best cognitive performance is achieved by a diet that is sufficiently rich in essential fatty acids, but as long as the omega-6:3 ratio is not high.

## Introduction

Honey bees (*Apis mellifera*) are social insects that live in highly organized colonies, consisting of a queen, many workers, and some drones. Division of labor among the workers is age-dependent (Winston, 1987). Young bees mostly work inside the colony, whereas older bees engage in foraging. Honey bee foraging behavior, as well as other characteristics of the honey bee, makes them the most important pollinator in commercial crops (Klein et al., 2007) providing important contributions to human nutrition (Chaplin-Kramer et al., 2014). Bees require floral nectar and pollen for their nutrition. Nectar is the main source of carbohydrates and pollen provides micro- and macro-nutrients, including proteins, fatty acids (FA), vitamins, and minerals. Bees prefer to collect pollen from a variety of plants (Avni et al., 2009). Moreover, colony performance is affected by the quality and quantity of pollen that the colony consumes and high lipid levels in pollen was found to promote honey bee health (Di Pasquale et al., 2013). Starvation and malnutrition were rated as the second main reason, after poor quality queens, for colony loss in the United States (Hayes et al., 2008). There is therefore growing interest in research of honey bee nutrition (Manning, 2016; Démares et al., 2017; Corby-Harris et al., 2018).

Fatty acid contents and composition in pollen varies between different types of plants (Roulston and Cane, 2000). In modern agriculture, beehives are frequently placed in large monoculture areas, where bees forage on single pollen. This may lead to a diet that is unbalanced in its essential components, such as amino and fatty acids, which could lead to malnutrition (Naug, 2009). Fatty acids are the main component in cell membranes and are important for their function. They are necessary for reproduction and development, serve as a source for energy and for the development of fat bodies in bees during winter (Kunert and Crailsheim, 1988; Manning, 2001).

Most fatty acids can be synthesized endogenously according to the body’s needs. Fatty acids that the body cannot produce must be provided through nutrition, accordingly they are called essential fatty acids (EFAs). Two groups of EFAs are omega-3 and omega-6, which are polyunsaturated fatty acids (PUFAs) (Simopoulos, 1991). Alpha-linolenic acid (ALA) and linoleic acid (LA) are the major omega-3 and omega-6 fatty acids, respectively, found in pollen, though their abundance differs between different pollen species (Manning, 2001). In mammals, both EFAs can be elongated to long chain PUFAs, LA to arachidonic acid (AA) and ALA to eicosapentaenoic acid (EPA) and docosahexaenoic acid (DHA). Those are the dominant EFAs in mammals and can be obtained in the diet mainly through fish oil and marine algae (Simopoulos, 2009).

Because of the low prevalence of omega-3 in the modern western diet, most researches have focused on the impact of deficiency of this FA. In mammals, the importance of omega-3 fatty acids is well known. These fatty acids constitute a major proportion of total FAs of brain, retina, and sperm in humans and other mammals (O’Brien et al., 1964). Deficiency of omega-3 fatty acids, mainly long-chain PUFAs, is associated with increase in frequency of chronic diseases, poor health and especially with several mental and cognitive disorders (Yashodhara et al., 2009; Gow and Hibbeln, 2014). The nutritional effects of deficiency in omega-3 in insects were investigated for the first time in honey bees (Arien et al., 2015). Bees that were fed the low omega-3 diets had great decrease in olfactory and tactile associative learning. These findings showed, that similar to mammals, omega-3 fatty acids have a crucial role for cognitive function of honey bees.

However, it is debated in the mammalian (including human) literature as to the relative detrimental effect of omega-3 deficiency as opposed to a high omega-6:3 ratio. The modern Western diet, for example, is biased toward omega-6, with omega-6:3 ratio of about 15:1, whereas this ratio in traditional human diets was about 1:1 (Simopoulos, 2009). In mammals, LA and ALA can be desaturated through enzymes to long chain PUFAs. Not only that this conversion process is very slow (Chow, 2000), but also there is competition between omega-6 and omega-3 fatty acids on the affinity to the desaturation enzymes. There are two enzymes (delta-4 and delta-6 desaturases) with greater affinity to omega-3 over omega-6 (Insua et al., 2003; Bazan, 2006). However, a high intake of LA interferes with the desaturation and elongation of ALA (Patterson et al., 2012).

A similar question arises in honey bee nutrition: whether bees require a particular absolute amount of omega-3 or to maintain a particular omega-6:3 ratio. Insects have only trace amounts of long-chain PUFAs (Shen et al., 2010). The dynamics between LA and ALA may be different than those in mammals. However, the findings of a very strong effect on bee cognition of ALA deficiency (unlike mammals in which cognitive impairment results from EPA and DHA deficiency), raises the hypothesis that there may be important LA:ALA dynamics in bees that affect bee cognition and health.

The primary aim of this research was to test whether the cognitive impairment in honey bees is due to low absolute amounts of omega-3 in the diet or to a high omega-6:3 ratio. In making diets that differed in these two factors, also the total lipid levels varied. A second aim, therefore, was to test the effect of total lipid levels on cognitive performance. Newly emerged bees were fed for a week diets that differed in omega 6:3 ratio and total lipids levels and were then tested in an olfactory conditioning test.

## Materials and Methods

### Diets

In order to control the fatty acids composition in the bees nutrition, we fed them artificial diets. As in previous experiments, we used soy flour as the source for protein (Arien et al., 2015). However, this flour also contains fatty acids, with the dominant one being omega-6 (LA), making it difficult to control omega-6:3 ratios. Therefore, we created diets based on flour after an oil extraction process, using a soxhlet system. Hexane at 70°C was used to extract residues of oil from the flour for 6 h, and was then evaporated to obtain fat-free flour. The protein contents of the soy flour was 47% protein (analyzed by the Kjeldahl method; see Arien et al., 2015), and was added as to achieve a 20% protein diet. The composition of the diets was: 42% fat-free soy flour, between 49.5 and 56.5% honey, which contains negligible amount of lipids (Machado De-Melo et al., 2018), and 1–8% mixture of two vegetable oils: flax and corn. Flax oil is 97% fatty acids, and is rich in omega-3, whereas corn oil is 90% fatty acids, and is rich in omega-6. The relative amount of each oil varied between treatments to achieve the desired FA composition (see Arien et al., 2015 for FA analyses of these oils). **Table 1** shows the EFA composition of the diet treatments. There were four groups of treatments with different levels of percentage of lipids in the diet: 1, 2, 4, and 8%, and within each group the ratio of omega-6 to omega-3 oils was 5, 1, or 0.3. The diets were designed so that we could compare the same three levels of omega-6:3 ratio in four levels of lipid concentration and with different absolute omega-3 amounts. We could thus compare the learning ability of bees fed diets that varied in omega-6:3 ratio but had similar absolute omega-3 amounts and we could compare bees fed diets that varied in absolute omega-3 amounts while maintaining the same omega-6:3 ratio.

**Table 1:** The experimental diets by their lipid percentage and omega-6:3 ratio, the essential fatty acid composition of the total fatty acids (TFA) and absolute amounts.

Omega 6:3 ratio	Lipids (%)	Omega 6 (% of TFA)	Omega 3 (% of TFA)	Omega 6 (mg/g Diet)	Omega 3 (mg/g Diet)
0.3	1	15.5	47.37	1.55	4.74
1	1	28.04	28.12	2.8	2.81
5	1	41.01	8.2	4.1	0.82
0.3	2	15.5	47.37	3.1	9.47
1	2	28.04	28.12	5.61	5.62
5	2	41.01	8.2	8.2	1.64
0.3	4	15.5	47.37	6.2	18.95
1	4	28.04	28.12	11.21	11.25
5	4	41.01	8.2	16.4	3.28
0.3	8	15.5	47.37	12.4	37.9
1	8	28.04	28.12	22.43	22.49
5	8	41.01	8.2	32.81	6.56

The essential FAs comprised between 54 and 65% of the TFA. The relative composition of the two essential FAs varied most, in comparison to the common non-essential FAs, between the different omega-6:3 ratio diets (Supplementary Table S1).

### Bees

Bees were of the local strain of honey bees, which is based mostly on the Italian bee, *Apis mellifera ligustica*. We placed sealed brood combs from ordinary hives in an incubator overnight. The following day we randomly collected up to 24 h-old bees that emerged in the incubator and placed them inside 9-cm petri-dishes with filter paper at the bottom, in groups of five bees. To each petri-dish we added two 1-ml Eppendorf feeders, one with diet and one with water. The bees were fed one of the diet treatments for 1 week, as in Arien et al. (2015); pollen consumption is mostly by young bees during the first days after emergence (Crailsheim et al., 1992). Diet consumption per dish was calculated by weighing the feeders at the beginning and after the 1 week in the incubator, taking into account weight loss due to evaporation by having for each diet a control dish with no bees. The diets contained honey so there was no need for supplemental carbohydrates. Then the bees were taken for olfactory conditioning of the proboscis-extension response (PER) experiments. There were between 31 and 34 bees in each treatment in PER experiments.

### Olfactory Proboscis-Extension Response (PER) Conditioning

Proboscis-extension response experiments were preformed according to established methods (Bitterman et al., 1983; Drezner-Levy et al., 2009). The experiment was conducted in a temperature-controlled laboratory with AC set at 26°C (range was 24–29°C). The petri-dish with the bees was placed in a freezer for 3–5 min, and then the immobilized bees were restrained into 5-cm long pieces of drinking straws by attaching duct tape around the sectioned top part of the straw and the thorax of the bee. Forty-five minutes later all bees were fed 1 μl of 50% (w/w) sucrose. One hour after the feeding we tested the bees for their appetitive motivation. We touched the antennae of each bee with a cotton stick soaked in 50% (w/w) sucrose solution; the bees were not fed during this test. Bees that did not extend their proboscis were removed from the experiment. Twenty motivated bees, which did extend their proboscis, were taken for conditioning and mounted along rulers in haphazard order. The experiment started immediately after the motivation test. The odors used in this experiment were Benzyl acetate and Geranyl acetate. To provide the odors we placed a strip of filter paper inside a glass syringe tube and dripped on it 3.5 μl of pure odor. The syringe was connected to an air pump controlled by computer. The odor was delivered for 4 s followed by provisioning of a reward for 3 s. The bees were exposed to the two odors in 12 conditioning trials, 6 to each odor, with an inter-trial interval of 8 min. One odor was associated with a positive reward (odor A) and the other odor with a negative reward (odor B). Odors were presented in a pseudorandom sequence ABBABAABABBA. Following presentation of odor A, the bees were fed by a Gilmont micro syringe 0.4 μl 50% sucrose solution as a positive reward (CS+). Following presentation of odor B, the negative reward (CS-) consisted of touching the antennae with a cotton-stick dipped in a 2 M NaCl solution (the bee was not fed the salt solution).

### Statistical Analyses

To test the effect of diets on learning performance we calculated a learning index, which was the difference between the sum of responses in the three last trials to the CS+ and CS- (Shafir and Yehonatan, 2014). We used a two-way ANOVA to test the effect of the omega 6:3 ratio and percentage of lipids in diets as main factors, their interaction and hive number as random variable, on the learning index. All statistics were done using the JMP v.13 software (SAS Institute).

## Results

**FIGURE 1:**
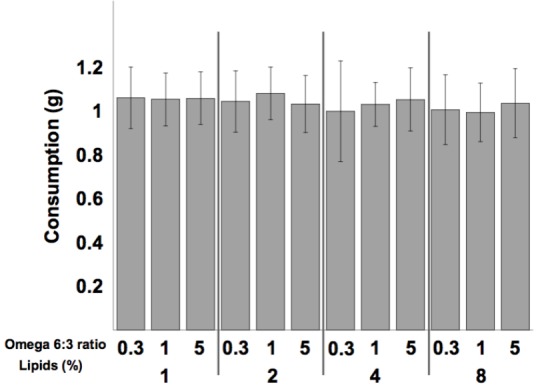
Mean (±SE) weekly consumption of the diets per dish (5 bees). Sample size equals 26 dishes for each of the 12 treatments.

Mean diet consumption per dish was not affected by the omega 6:3 ratio (*F*_2,300_ = 0.37, *P* = 0.69), nor by the total lipid concentration (*F*_3,300_ = 1.603, *P* = 0.19), and the interaction between these two factors was not statistically significant (*F*_6,300_ = 0.6, *P* = 0.73). Comparison of the weekly diet consumption between all 12 treatments is shown in **Figure 1**.

**FIGURE 2:**
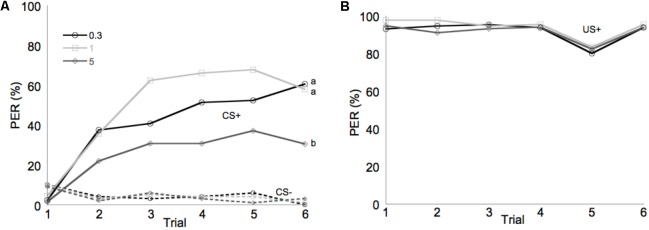
Performance in olfactory conditioning of bees fed diets with omega-6:3 ratio of 0.3 (*N* = 133), 1 (*N* = 132), or 5 (*N* = 136). Data are pooled for all four total lipid concentrations tested. **(A)** Learning curves show the proportion of bees that extended their proboscis to the conditioned odors in each of six trials with each odor. The full lines show learning curves to a positively rewarded conditioned stimulus (CS+). The dashed lines represent response to the negatively rewarded conditioned stimulus (CS–). **(B)** Shows the response to the sucrose reward, unconditioned stimulus (US+). Different letters represent statistically significant difference between treatments (Tukey HSD test, *P* < 0.05).

**FIGURE 3:**
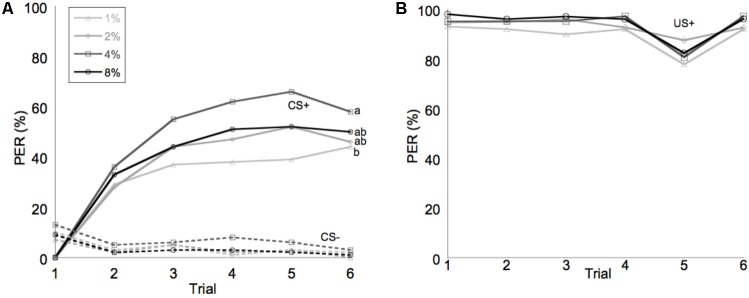
Average of performance in olfactory conditioning of bees according to percentage of lipids in the diet. **(A)** Learning curves show the proportion of bees that extended their proboscis to the conditioned odor in each of six trials with each odor. The full lines show learning curves to a positively rewarded conditioned stimulus (CS+). The dashed lines represent the negative rewarded conditioned stimulus (CS-). **(B)** Shows the response to the sucrose reward, unconditioned stimulus (US+). Different letters represent statistically significant difference between treatments (Tukey HSD test, *P* < 0.05).

**FIGURE 4:**
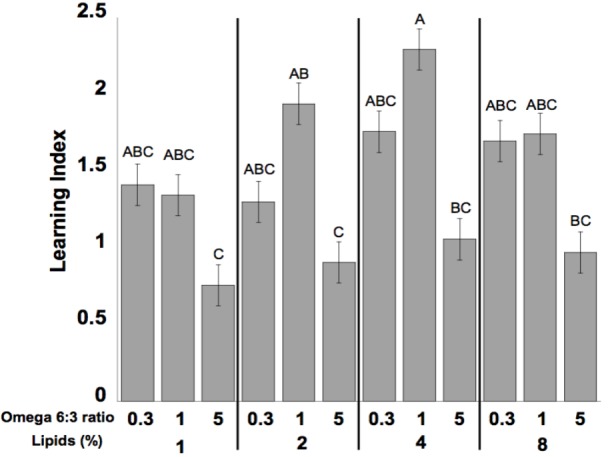
Mean of learning index of the bees for each of the different diets. The learning index for each bee is the difference between the sum of responses during the last three trials to the positively rewarded (sugar) odor (CS+) and the sum of responses during the last three trials to the negatively rewarded (salt) odor (CS-). The numbers at the bottom of bars represent sample size. Different letters represent statistically significant difference between treatments (Tukey’s test, *P* < 0.05).

Learning performance was significantly affected by diet omega-6:3 ratio (*F*_2,386_ = 17.9, *P* < 0.0001) and total lipid concentration (*F*_3,377.5_ = 2.96, *P* = 0.03), with the interaction between the two factors not being significant (*F*_6,385_ = 0.79, *P* = 0.58). **Figure 2A** shows the learning curves of bees fed diets that differed in omega-6:3 ratio, pooling together all total lipid concentrations. Such comparison shows that learning performance of bees fed diets with omega-6:3 ratio of 5 was significantly lower than of those fed diets with lower omega-6:3 ratios of 1 and 0.3. When presented with sucrose solution, almost all bees imbibed it in almost all the trials, and there was no difference in the US response between groups (*F*_2,386_ = 0.04, *P* = 0.96) (**Figure 2B**). **Figure 3A** shows the learning curves of bees fed diets that differed in total lipid concentration, pooling together all omega-6:3 ratios. Lowest performance was of bees fed diets with 1% lipids, and best performance was of bees fed diets with 4% lipids. The percentage of lipids in diets also did not affect the US response (*F*_3,377.5_ = 0.36, *P* = 0.78) (**Figure 3B**). Comparison of learning indexes between all 12 treatments is shown in **Figure 4**. The learning index of bees fed omega-6:3 ratio of 5 was consistently the lowest within all lipids groups. Bees which had omega-6:3 ratio of 1 in their diets with 4 and 2% lipids, achieved the highest learning scores (**Figure 4**).

## Discussion

In the present paper, we study the effect of omega-6:3 ratio and lipids content in honey bee nutrition on learning ability. Our results are consistent with our previous findings (Arien et al., 2015), that learning performance in bees is greatly impaired by a diet deficient in omega-3 and high in omega-6:3 ratio. Here, however, we experimentally separated the effect of absolute levels of omega-3 from that of the omega-6:3 ratio. We found that a minimal total absolute amount of EFAs is required, but thereafter the main effect on learning performance is of the omega-6:3 ratio. Specifically, high omega-6:3 ratio impairs learning, even if the absolute amount of omega-3 in the diet is relatively high. Bees fed diets that had lower omega-6:3 ratio (1 or 0.3) learned better than those fed diets with high omega-6:3 ratio (of 5), even when the absolute amount of omega-3 was similar.

In human nutrition, a high omega-6:3 ratio has been associated with cognitive decline in adults (Loef and Walach, 2013). The amount of omega-6 consumed can modulate the amount of omega-3 FAs and thereby reduce the amount of omega-3 available in the body (Taha et al., 2014). Adults that performed poorly in cognitive tests had higher ratio of omega-6:3 FAs in their blood plasma compared to those that performed better (Cherubini et al., 2007).

Andruchow et al. (2017) found a correlation in healthy older adults between dietary ratio of omega-6:3 and spatial cognition; those whose diet contained a lower omega-6:3 ratio had better spatial memory and performed better in navigation tests. Similarly, rats fed lower omega-6:3 ratio diets performed better in a task requiring navigating a maze (Hajjar et al., 2012). Similarities between bees and mammals in the detrimental effects of high dietary omega-6:3 ratio on learning performance suggests that similar to mammals, also bee spatial cognition might be impaired by high omega-6:3 ratio. Honey bees have sophisticated navigation and orientation abilities, which are crucial for colony survival (Collett et al., 2013). Colony collapse disorder (CCD), for example, involves bees departing the colony and failing to return to if for yet unknown reasons (Oldroyd, 2007; Traynor et al., 2017). The effect of high omega-6:3 ratio diet on honey bee navigation and spatial learning deserves further study.

In a study in which pollen was collected by hand from 28 different plant species, the range of omega-6:3 ratio was between 0.09 and 5.34 (Arien et al., 2015). The highest values were of *Eucalyptus* trees. Our results suggest that a colony situated in a *Eucalyptus* monoculture forest would suffer from this high omega-6:3 ratio. There are other crops that are grown in monocultures, and which have relatively high omega-6:3 ratio pollen, which are probably not ideal for a colony. However, when a colony is situated in a habitat with diverse vegetation, it tends to collect pollen from several plants at the same time (Avni et al., 2009). The omega-6:3 ratio of pollen mixtures collected by bees in several places around the world ranged between 0.3 and 0.9 (Arien et al., 2015), which is in the optimal range for cognitive functions according to our results.

It is debated whether honey bee foragers can assess the nutritional value of pollen, especially with regards to its protein contents (reviewed by Zarchin et al., 2017). However, in choice experiments, Hendriksma and Shafir (2016) recently showed that honey bee foragers preferred to collect diets that balanced their nutritional deficiencies, including in essential amino acids and EFAs. Furthermore, when a colony was fed pollen lacking a specific EFA, foragers attempted to compensate for this deficiency at the colony level by evaluating complementary pollen as more attractive in their recruitment dances (Zarchin et al., 2017). Thus, it appears that a honey bee colony needs a balanced omega-6:3 diet, and that it attempts to selectively forage so as to achieve it. The geometric framework approach to nutrition (Simpson and Raubenheimer, 2012) has been applied lately to assess the macronutrient requirements of honey bees, for example the balance between proteins and carbohydrates (Paoli et al., 2014; Helm et al., 2017). We are presently using this approach to further evaluate the omega-6:3 requirements of honey bees.

Bumblebee foragers prefer a protein to lipids (P:L) ratio of between 5:1 and 10:1 (Vaudo et al., 2016a,b). Interestingly, callow honey bees in our study consumed equal amounts of all diets (**Figure 1**), though the P:L ratio of our diets ranged between 20:1 and 2.5:1, for the 1 and 8% lipid diets, respectively. These young bees, during the first week of their life, are the main consumers of pollen in the colony (Crailsheim et al., 1992). It appears that young honey bees, until the age of 1 week, may be focused on protein and not be regulating lipid consumption.

Learning performance was affected also by the diet lipids content; bees fed a low-fat diet of 1% lipids had the lowest learning curve, regardless of the omega-6:3 ratio. Thus, even a diet whose EFA contents was strongly biased toward omega-3 (with omega-6:3 ratio of 0.3), could not support good learning when the total lipid content (and therefore the absolute amount of omega-3) was too low. The reported range of pollen lipids content is between 2 and 20%; the range is reduced to between 3 and 8% for bee bread, which consists of a mixture of several pollens stored in cells within the hive (Wright et al., 2017). In this study, we show that for good learning ability lipid levels should be between 2 and 8% with peak performance at 4% total lipids. Relatively high pollen lipid concentration is also important for proper brood development. Di Pasquale et al. (2013) found that young nurse bees developed well when fed several pollens with total lipid contents of between 6.4 and 7.4%.

Impairment of olfactory associative learning may have direct adverse consequences to the functioning of a honey bee colony (Klein et al., 2017). In the present study, we tested the effect of a pollen-substitute diet on the performance of 8-day-old bees. The typical task of young bees at this age is to be nurses, which attend to and feed the larvae (Page and Robinson, 1991). Many of the social interactions between adult bees and between nurse bees and larvae depend on chemical signaling (Amdam, 2011). We have previously shown that the impaired learning performance due to omega-3 deficiency could not be attributed solely to impairment of olfactory perception, as tactile associative learning was equally affected (Arien et al., 2015). We are currently testing specifically whether olfactory perceptual abilities are also affected by omega-6:3 imbalances. It remains to be determined how impairment of olfactory perception and/or of olfactory associative learning would impact the ability of nurse bees to raise larvae.

Since older bees hardly consume pollen any more, we assume that the detrimental cognitive effects accumulated over the first week of life would persist into older age. We in fact found severe learning deficits in older bees from a colony fed an omega-3 deficient diet (Arien et al., 2015). The typical task of older bees is foraging, a task that requires sophisticated cognitive abilities. Foragers need to quickly learn to associate between floral attributes and nectar and/or pollen rewards (Menzel, 1999), and between floral attributes and predation risk (Abbott and Dukas, 2009). Thus, the learning impairments conferred by nutritional deficits of callow bees is likely to adversely affect the foraging behavior and survival of older bees.

Honey bees have provided an exceptionally rich model for comparative cognition (Menzel, 2012; Giurfa, 2015; Perry et al., 2017). We have previously shown that as in mammals, omega-3 deficiency severely impaired honey bee associative learning (Arien et al., 2015). Here, we strengthen this finding and furthermore show that, as hypothesized for mammals, learning performance is mostly affected by dietary omega-6:3 ratio. The honey bee may prove a useful model for comparative studies of the nutritional basis of cognitive performance.

## Author Contributions

YA, AD, and SS designed the experiments. YA and SS wrote the paper with help from AD and analyzed the data. YA performed the experiments.

## Conflict of Interest Statement

The authors declare that the research was conducted in the absence of any commercial or financial relationships that could be construed as a potential conflict of interest. The reviewer ML and handling Editor declared their shared affiliation.
