# Supplementary material for: Omega-6:3 Ratio More Than Absolute Lipid Level in Diet Affects Associative Learning in Honey Bees
Source: Front Psychol. 2018 Jun 19;9:1001. doi: 10.3389/fpsyg.2018.01001 (PMC6018467; doi:10.3389/fpsyg.2018.01001)
Supplement: Supplementary file 1 [file Table_1.DOCX]

**Table S1.** FA composition (% of TFA) in the diets of the three omega-6:3 ratios. The essential FAs are alpha-linolenic acid (ALA, omega-3) and linoleic acid (LA, omega-6). The more common non-essential FAs are palmitic acid (PA), stearic acid (SA), and oleic acid (OA).

| **Omega-6:3 ratio/FA** | **0.3** | **1** | **5** |
| --- | --- | --- | --- |
| C14:0 | 0.04 | 0.03 | 0.03 |
| C16:0 (PA) | 5.98 | 8.11 | 10.47 |
| C16:1 n-7 c | 0.05 | 0.07 | 0.08 |
| C18:0 (SA) | 5.11 | 4.08 | 2.95 |
| C18:1 n-9 t | 0.02 | 0.02 | 0.02 |
| C18:1 n-9 c (OA) | 22.20 | 25.84 | 29.85 |
| C18:1 n-7 t | 0.81 | 0.76 | 0.70 |
| C18:2 n-6 t | 0.01 | 0.02 | 0.02 |
| C18:2 n-6 c (LA) | 15.91 | 29.72 | 45.00 |
| C18:3 n-6 c | 0.01 | 0.01 | 0.01 |
| C18:3 n-3 c (ALA) | 48.62 | 29.80 | 8.99 |
| C20:0 | 0.25 | 0.44 | 0.65 |
| C20:1 n-9 c | 0.24 | 0.29 | 0.35 |
| C20:2 n-6 c | 0.15 | 0.11 | 0.06 |
| C20:3 n-6 c | 0.01 | 0.02 | 0.02 |
| C22:0 | 0.26 | 0.27 | 0.28 |
| C22:1 n-9 c | 0.01 | 0.02 | 0.03 |
| C23:0 | 0.05 | 0.05 | 0.05 |
| C24:0 | 0.22 | 0.31 | 0.40 |
| C24:1 n-9 c | 0.04 | 0.03 | 0.03 |
